# Salivary Leucocytes as In Vitro Model to Evaluate Nanoparticle-Induced DNA Damage

**DOI:** 10.3390/nano11081930

**Published:** 2021-07-27

**Authors:** Vanessa Valdiglesias, Natalia Fernández-Bertólez, Carlota Lema-Arranz, Raquel Rodríguez-Fernández, Eduardo Pásaro, Ana Teresa Reis, João Paulo Teixeira, Carla Costa, Blanca Laffon

**Affiliations:** 1 Universidade da Coruña, Grupo DICOMOSA, Centro de Investigaciones Científicas Avanzadas (CICA), Departamento de Biología, Facultad de Ciencias, Campus A Zapateira s/n, 15071 A Coruña, Spain; carlota.lema.arranz@udc.es; 2 Instituto de Investigación Biomédica de A Coruña (INIBIC), AE CICA-INIBIC, Oza, 15071 A Coruña, Spain; natalia.fernandezb@udc.es (N.F.-B.); Raquel.rodriguez4@udc.es (R.R.-F.); eduardo.pasaro@udc.es (E.P.); blaffon@udc.es (B.L.); 3 Universidade da Coruña, Grupo DICOMOSA, Centro de Investigaciones Científicas Avanzadas (CICA), Departamento de Psicología, Facultad de Ciencias de la Educación, Campus Elviña s/n, 15071 A Coruña, Spain; 4 Environmental Health Department, National Institute of Health, 4000-055 Porto, Portugal; anateresareis@gmail.com (A.T.R.); jpft12@gmail.com (J.P.T.); carla.trindade@insa.min-saude.pt (C.C.); 5 EPIUnit-Instituto de Saúde Pública, Universidade do Porto, 4050-600 Porto, Portugal; 6 Laboratory for Integrative and Translational Research in Population Health (ITR), 4050-600 Porto, Portugal

**Keywords:** TiO_2_ nanoparticles, ZnO nanoparticles, CeO_2_ nanoparticles, salivary leukocytes, comet assay, interference, genotoxicity

## Abstract

Metal oxide nanoparticles (NPs) have a wide variety of applications in many consumer products and biomedical practices. As a result, human exposure to these nanomaterials is highly frequent, becoming an issue of concern to public health. Recently, human salivary leucocytes have been proposed as an adequate non-invasive alternative to peripheral blood leucocytes to evaluate genotoxicity in vitro. The present study focused on proving the suitability of salivary leucocytes as a biomatrix in the comet assay for in vitro nanogenotoxicity studies, by testing some of the metal oxide NPs most frequently present in consumer products, namely, titanium dioxide (TiO_2_), zinc oxide (ZnO), and cerium dioxide (CeO_2_) NPs. Primary and oxidative DNA damage were evaluated by alkaline and hOGG1-modified comet assay, respectively. Any possible interference of the NPs with the methodological procedure or the hOGG1 activity was addressed before performing genotoxicity evaluation. Results obtained showed an increase of both primary and oxidative damage after NPs treatments. These data support the use of salivary leucocytes as a proper and sensitive biological sample for in vitro nanogenotoxicity studies, and contribute to increase the knowledge on the impact of metal oxide NPs on human health, reinforcing the need for a specific regulation of the nanomaterials use.

## 1. Introduction

The fast development of the nanotechnology industry has resulted in a growing public debate on the possible health impact of the new engineered nanoproducts. Together with the direct exposure of subjects involved in nanomaterial manufacturing processes or patients subjected to nanomedical procedures and/or treatments, these new products can be released into the environment and appear in water, air, and soil, becoming environmental pollutants and entailing a potential threat for both humans and ecosystems [1]. Consequently, the risk assessment of nanomaterials entering the environment and present in workplaces is becoming an increasingly important issue for governments, regulatory authorities, and international organizations that work in developing policy frameworks and guidance documents related to nanotechnology and nanosafety [2,3]. A key advance on this topic has been recently made to provide clear guidance on aquatic toxicity testing of manufactured nanomaterials using OECD methods [4]. Still, despite this common global effort, there is an important lack of toxicological information that makes it difficult to move forward in these policies [3,5]. A deep understanding of the toxicological profile of these novel engineered nanomaterials is necessary in order to develop a proper regulatory context ensuring that these compounds are safe for use and are produced responsibly, with optimization of benefits and minimization of risks. The lack of adequate detection and characterization techniques and of reproducible and validated methods for toxicological assessment of nanomaterials have been identified as major bottlenecks for the safe and sustainable use of nanomaterials [6].

Metal-based is one of the five categories in which nanomaterials are classified, and includes metal(loid) oxides [7]. These are chemical compounds that contain at least one metal atom and one or more oxygen atoms. Among nanomaterials, metal oxide nanoparticles (NPs) have received considerable attention largely due to their variety of uses, namely in optics and electronics, healthcare, construction, automotive and personal care products [8]. Due to this wide diversity of applications, they are commonly present in many consumer products and biomedical practices [8,9]. As a result, human exposure to metal oxide NPs is highly frequent, becoming a matter of special concern for public health in the last years.

Due to the well-established link between DNA alterations and many pathologies and health conditions, genotoxicity of nanomaterials and, in particular, of metal oxide NPs, becomes an important issue that needs to be addressed and investigated providing data crucial for a complete risk assessment of these materials before mass production. However, while the acute toxicity of nanomaterials has been relatively widely evaluated, their genotoxicity has been less studied and the available information is still limited [10].

Together with the micronucleus test and the Ames test, the comet assay is the most frequently used approach for nanogenotoxicity evaluation [11]. The comet assay, also known as the single-cell gel electrophoresis assay, is a method for measuring DNA strand breaks in eukaryotic individual cells. Protocol variants also allow the specific evaluation of oxidative DNA damage, DNA methylation or DNA repair alterations, among other genetic insults [12]. Due to this versatility, together with its simplicity and sensitivity, this technique has been extensively employed in in vitro, in vivo and in human population studies using a number of different cell systems and biomatrices. Still, the most common human primary cell type used for in vitro studies and for biomonitoring studies are, by far, leucocytes collected from peripheral blood.

Recently, human salivary leucocytes have been proposed as a non-invasive adequate alternative to peripheral blood leucocytes to evaluate the genotoxic effects of recent exposure to environmental contaminants, particularly those involving inhalatory or oral exposure routes [13]. Moreover, this cell type might be also useful for in vitro testing, and indeed our group has recently proved that leucocytes isolated from saliva samples, both fresh and cryopreserved, can be employed as appropriate biomatrices to detect primary and oxidative DNA damage caused by different mechanisms by the comet assay [14]. However, their suitability and the specific protocol to be employed for in vitro nanogenotoxicity screening have not been described yet. 

On this basis, the present study focused on proving the suitability of human salivary leucocytes to be employed as biomatrix in the comet assay for in vitro nanogenotoxicity studies, by evaluating the potential genotoxicity of some of the metal oxide NPs most frequently present in consumer products, namely titanium dioxide (TiO_2_), zinc oxide (ZnO), and cerium dioxide (CeO_2_) NPs. Both primary and oxidative DNA damage evaluation were addressed by means of the alkaline standard and hOGG1-modified versions of the comet assay, respectively, previously checking the possible interference of the NPs with these procedures.

## 2. Materials and Methods

### 2.1. Chemicals

Titanium dioxide nanoparticles (TiO_2_ NPs) (rutile:anatase 15:85) (CAS No. 13463-67-7) was purchased from Degussa-Evonik (Bitterfeld, Germany). Zinc oxide nanoparticles (ZnO NPs) (CAS No. 1314-13-2), cerium dioxide nanoparticles (CeO_2_ NPs) (CAS No. 1306-38-3), dimethyl sulfoxide (DMSO) ACS reagent ≥99.9% (CAS number 67-68-5), Histopaque^®^-1077 sterile-filtered, methyl methanesulfonate (MMS) 99% (CAS number 66-27-3), potassium bromate (KBrO_3_) for analysis EMSURE^®^ Reag. Ph Eur (CAS number 7758-01-2), 4′,6-diamidine-2′-phenylindole (DAPI) (CAS No. 28718-90-3), and trypan blue (CAS No. 72-57-1) were purchased from Sigma-Aldrich Co (Madrid, Spain). Human 8-oxoguanine DNA glycosylase (hOGG1) was obtained from Trevigen Inc (Gaithersburg, MD, USA). MMS and KBrO_3_ were dissolved in sterile distilled water (dH_2_O).

### 2.2. Nanoparticle Suspensions: Preparation and Characterization

TiO_2_, ZnO and CeO_2_ NPs were suspended in either deionized water or leucocyte culture medium [RPMI 1640 medium containing 15% fetal bovine serum (FBS), 1% L-glutamine (200 mM), and 1% penicillin (5000 U/mL)/streptomycin (5000 μg/mL) (all from Gibco, Thermo Fisher Scientific Inc., Uppsala, Sweden)] at final concentrations of 150, 40 and 10 µg/mL, respectively, and ultrasonicated with a 2.5 mm probe (Sonoplus mini 20, Bandelin, Berlin, Germany) at 30 W for 5 min (0.5 min on and 1 min off twice, plus 2 min on). Three independent replicates of each suspension were performed. To avoid heating of the NPs suspensions, they were maintained in ice during the whole sonication procedure. Dynamic Light Scattering (DLS) and zeta potential measurements were performed with a Zetasizer Nano-ZS (Model ZEN 3600, Malvern Instruments, Worcestershire, UK), equipped with 4.0 mW 633 nm He-Ne laser. The DLS and laser Doppler velocimetry (LDV) were used for characterization of hydrodynamic diameter and charge (zeta potential) of the NPs, respectively.

### 2.3. Sample Collection and Processing

Saliva samples were obtained from 6 non-smoking healthy volunteers (3 women and 3 men, aged between 18 and 53). This study followed ethical criteria established by the Helsinki declaration and was approved by the University of A Coruña Ethics Committee (2021-0027). Prior to joining the study, each donor signed an informed consent. Donors were asked not to eat or drink anything but water in the hour preceding sampling. Saliva samples were collected from each subject by performing four consecutive mouth rinses (1 min each) with 10 mL of 0.9% NaCl sterile solution. The four rinses were combined in sterile 50 mL centrifuge tubes and centrifuged for 15 min at 1100× *g* and 4 °C. The supernatants were discarded, and cell pellets were re-suspended in 2 mL phosphate-buffered saline solution (PBS).

A pool was prepared combining cell suspensions from all donors in order to avoid results modifications due to interindividual variability; it was centrifuged for 15 min at 4 °C and 1100× *g*, and the cell pellet was re-suspended in 8 mL RPMI 1640 medium. Leucocytes were then isolated by density gradient centrifugation using Histopaque^®^-1077 (Sigma-Aldrich Co, Madrid, Spain), according to the manufacturer’s guidelines. In brief, cell suspension was carefully layered over 4 mL Histopaque^®^-1077, and centrifuged at 400× *g* for 30 min with brake turned off. The interface containing the leucocytes was collected and transferred to another tube with 5 mL PBS, and centrifuged for 15 min at 1100× *g*. The cell pellet was re-suspended at 2.5 × 10^6^ cells/mL in freezing medium [40% RPMI 1640, 50% FBS, and 10% DMSO], aliquoted into cryogenic vials, and stored at −80 °C for at least one week.

Additionally, a positive reference standard was prepared with salivary leucocytes obtained from the same six donors. The purpose of this reference standard was to control for interassay variability in the comet assay. Samples were collected and leucocytes were isolated as described above. After pooling all cell suspensions and centrifuging for 15 min at 1100× *g*, leucocytes were re-suspended at 1.56 × 10^6^ cells/mL in culture medium and treated with 50 µg/mL MMS (1% of final volume) for 3 h at 37 °C. Then, cells were centrifuged again, suspended in freezing medium, aliquoted at 1.3 × 10^5^ cells/mL into cryogenic vials, and stored at −80 °C. Leucocytes from one aliquot were used as reference standard in each experiment conducted.

### 2.4. Treatments

Frozen saliva leucocytes were quickly thawed at 37 °C, centrifuged at 1100× *g* for 15 min, and washed again with PBS. Aliquots containing 6.6 × 10^4^ cells/mL were treated at 37 °C for 3 h in culture medium containing each of the NPs at three different concentrations: 80, 120 and 150 µg/mL TiO_2_ NPs; 20, 30 and 40 µg/mL ZnO NPs, and 0.5, 2 and 10 µg/mL CeO_2_ NPs. The doses and treatment time were selected on the basis of previous studies [15,16,17]. Negative controls (culture medium) were included in each experiment. Positive controls used were 50 µg/mL MMS for 3 h in the case of primary DNA damage, and 335 µg/mL KBrO_3_ for 1 h in the case of oxidative DNA damage [14]. Trypan blue exclusion technique was used to assess cell viability after treatments. Thus, cells were centrifuged at 1100× *g* for 5 min, resuspended in 500 µL PBS, and 20 µL of the cell suspension was then mixed with 20 µL of 0.4% trypan blue solution. The number of trypan blue positive cells (not viable) was measured using a Neubauer cell counting chamber at a Nikon HFX-DX light microscope (Tokyo, Japan). Cell viability was determined as follows: %viability = 100 − [(number of dead cells/total number of cells counted) × 100], resulting higher than 80% in all cases.

### 2.5. Alkaline Comet Assay

Comet assay was described in this manuscript according to the Minimum Information for Reporting Comet Assay procedures and results (MIRCA) recommendations [18]. Primary DNA damage, i.e., single and double strand breaks, and alkali-labile sites, induced in salivary leucocytes by NPs exposure, was evaluated by the standard alkaline comet assay. At the end of the NPs treatments, cells were centrifuged for 3 min at 8700× *g*. Supernatant was removed, and 20 µL of the remaining cell suspension were mixed with 80 µL of freshly made 0.9% low-melting-point (LMP) agarose (final agarose concentration 0.72%). Two drops of 40 µL each of this suspension were placed on a slide previously coated with a layer of 1% normal-melting-point agarose, and covered with 20 × 20 mm coverslips. Agarose solidification was allowed for 15 min placing the slides on ice. Then, the coverslips were removed and the slides were immersed in lysis solution (250 mM NaOH, 10 mM Tris-HCl, 100 mM Na_2_EDTA, 2.5 M NaCl, pH 10, and 1% Triton X-100 added just before use) at 4 °C overnight in the dark to avoid additional DNA damage due to laboratory light conditions when handling photosensitizing compounds such as TiO_2_ [19]. After lysis, slides were placed on a horizontal electrophoresis tank in an ice bath, and incubated for 20 min in the dark in freshly prepared alkaline electrophoresis solution (300 mM NaOH, 1 mM Na_2_EDTA, pH > 13) for DNA unwinding. Electrophoresis was then conducted for 20 min at 0.83 V/cm. Slides were washed three times (5 min each) with neutralizing solution (0.4 M Tris–HCl, pH 7.5), and air-dried at room temperature in the dark. Preparations were stained with 25 µL of 5 µg/mL DAPI per drop, for at least 30 min before scoring. Slides were stored in a humidified sealed box at 4 °C to prevent drying of the gel. They were analyzed within six days.

Comet IV software (Perceptive Instruments, Suffolk, UK) was used for image capture and analysis. Fifty cells per drop (100 from each slide) were scored “blindly” by the same scorer using a magnification of 40×. Percentage of DNA in the comet tail (%tDNA) was used as a DNA damage parameter.

Positive reference standards (assay controls) were used in all experiments in order to correct sample data for experimental variation. The correction factor for normalization was calculated according to Collins et al. [20]. In all experiments, this correction factor ranged between 0.98 and 1.05.

Additionally, prior to conducting the comet assay experiments, the possible interference of the NPs with the comet assay protocol was tested following Magdolenova et al. [21]. Briefly, after thawing, leucocytes were centrifuged for 3 min at 8700× *g*, supernatant was removed, and 20 µL of the cell suspension were mixed with 40 µL of 1.8% LMP agarose and 40 µL of each NPs so that their final concentration was the highest tested in this study (150, 40, and 10 µg/mL for TiO_2_, ZnO and CeO_2_ NPs, respectively). Two drops of 40 µL each of this suspension were used to prepare a slide. Then, the alkaline comet assay was performed according to the general protocol described above. Results obtained for each type of NPs were compared with a negative control (leucocytes without NPs).

### 2.6. hOGG1-Modified Comet Assay

In order to evaluate oxidative DNA damage, the hOGG1-modified comet assay was conducted at the end of NPs treatments. Duplicate slides were prepared for each experimental condition (two agarose drops per slide). After the lysis step in the standard alkaline comet assay, slides were washed with enzyme buffer (0.1 M KCl, 0.5 mM EDTA, 40 mM Hepes, 0.2 mg/mL bovine serum albumin, pH 8.0) 3 times for 5 min each. One of the slides from each condition was treated with 50 μL hOGG1 (0.0016 U/μL buffer), and the other slide was treated with just 50 μL of enzyme buffer. Coverslips were placed on top of each drop, and slides were incubated at 37 °C for 10 min in a humidified box. Slides were then processed as in the standard alkaline comet assay described above. Net hOGG1-sensitive sites were calculated for each experimental condition by subtracting the %tDNA corresponding to the buffer incubation from that corresponding to the enzyme incubation. All experiments included positive reference standards; normalization was conducted following Collins et al. [20].

Possible interference of the NPs with hOGG1 enzyme activity was tested previous to the hOGG1-modified comet assay experiments, according to the indications of Magdolenova et al. [21]. In brief, leucocytes were incubated for 1 h either with 335 µg/mL KBrO_3_ plus each NPs at the highest concentration tested, or with just KBrO_3_. Then, hOGG1-modified comet assay was performed as described above. Results obtained for each type of NPs+KBrO_3_ and for KBrO_3_ were compared with a negative control (cells without any treatment).

### 2.7. Statistical Analysis

Experimental data were expressed as mean ± standard error. Three independent experiments were carried out for each experimental condition tested, and each condition was always run in duplicate. Kruskal–Wallis test and Mann–Whitney *U*-test were used to analyze differences among groups (all concentrations tested under the same conditions) and two-by-two comparisons, respectively. Concentration–response relationships were assessed by Spearman’s correlation. The threshold of significance was set at 0.05. Statistical analyses were performed using SPSS for Windows statistical package V. 21.

## 3. Results

In this study, salivary leucocytes were tested as in vitro model for evaluating nanomaterials genotoxicity. Cells were exposed to three different metal oxide NPs, and DNA damage induction, both primary and oxidative, was investigated by means of comet assay-based methodologies. The possible interference of the NPs with the comet assay procedure and hOGG1 enzyme activity was checked prior the experiments. Frozen cells were used since a recent study demonstrated that cryopreserved salivary leucocytes show similar sensitivity to DNA damage induction by genotoxic agents than fresh cells and, consequently, both can be equally employed as appropriate biomatrices to detect DNA damage by the comet assay [14].

### 3.1. Nanoparticle Characterization

Table 1 summarizes the main physical–chemical properties of the NPs tested, namely size, hydrodynamic diameter, and zeta potential, suspended in water and in leucocyte culture medium. The size distribution graphs obtained showed a good dispersion of the NPs in all cases (data not shown). Slightly higher values of the mean hydrodynamic diameter were observed in culture medium than in aqueous suspensions for TiO_2_ and CeO_2_ NPs, but the value obtained in medium for ZnO NPs was notably lower than the one in water. On the contrary, values of zeta potential were negative and lower (in absolute value) in medium than in water, except for CeO_2_ NPs, that showed a mildly lower positive value in water.

### 3.2. Primary DNA Damage

In order to avoid false positive results in the genotoxicity assessment due to cytotoxicity, cell viability was evaluated after NPs treatments. Values obtained for the highest NPs concentrations tested were always higher than 80% (86.0 ± 2.0% for TiO_2_ NPs, 84.84 ± 2.9% for ZnO NPs, and 86.7 ± 2.2% for CeO_2_ NPs).

**Figure 1:**
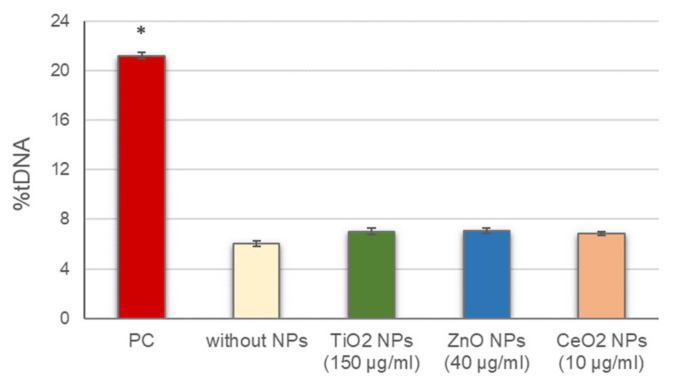
Assessment of NPs interference with the comet assay methodology. Columns represent mean values and error bars indicate the standard errors. PC: positive control (50 µg/mL MMS for 3 h). * *p* < 0.01, significant difference with regard to without NPs.

Before conducting the standard alkaline comet assay experiments, the potential interference of NPs with this methodology, including alterations of DNA migration during electrophoresis, either by inducing breaks into the naked DNA or inhibiting DNA migration, was assessed. No significant differences in DNA breaks were observed between cells in the presence of the different NPs, added to the agarose just before preparing the gels, and cells in the absence of NPs (Figure 1). These results indicate that the presence of NPs did not interfere with the normal development of the assay and the DNA damage detection.

**Figure 2:**
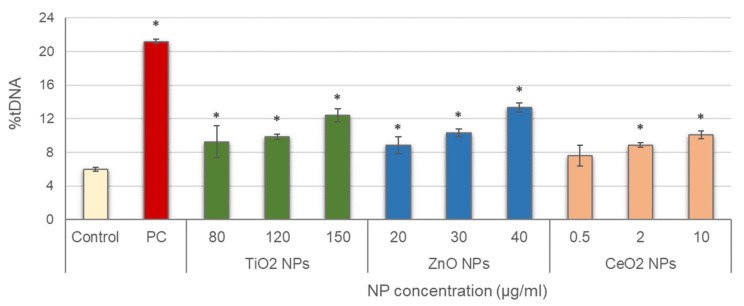
Results of primary DNA damage induced in salivary leucocytes exposed to TiO_2_, ZnO and CeO_2_ NPs. PC: positive control (50 µg/mL MMS for 3 h). Columns represent mean values and error bars indicate the standard errors. * *p* < 0.01, significant difference with regard to the negative control.

Leucocytes were then exposed to the three NPs, and alkaline comet assay was conducted; results are depicted in Figure 2. Significant increases in DNA damage with regard to the negative control were obtained in all treatments tested, with concentration-dependent increasing trends for all NPs (TiO_2_ NPs: r = 0.953; *p* < 0.01; ZnO NPs: r = 0.969; *p* < 0.01; CeO_2_ NPs: r = 0.926; *p* < 0.01).

### 3.3. Oxidative DNA Damage

**Figure 3:**
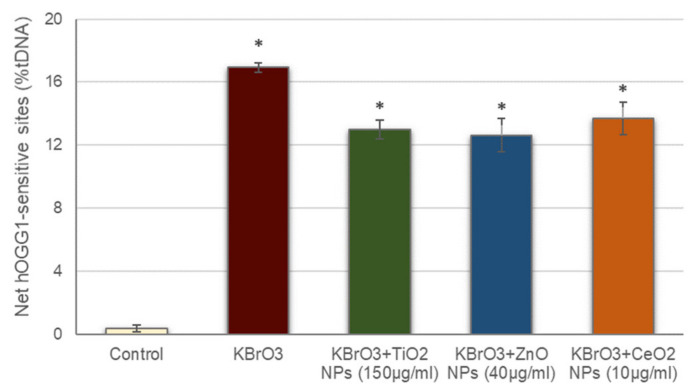
Assessment of NPs interference with hOGG1 enzyme activity. Columns represent mean values and error bars indicate the standard errors. * *p* < 0.01, significant difference with regard to the control.

Prior to addressing oxidative DNA damage induction by the studied NPs, the possible interference of these NPs with the hOGG1 enzyme activity was also assessed (Figure 3). The net hOGG1-sensitive sites observed in cells treated with KBrO_3_ in the presence of the different NPs was significantly increased with regard to the negative control, similarly to what occurred with cells treated with just KBrO_3_. These data demonstrate that hOGG1 enzyme in the presence of the NPs is still able to detect the oxidative DNA damage induced by KBrO_3_, proving that NPs presence did not interfere with the ability of hOGG1 to effectively detect oxidized DNA bases.

**Figure 4:**
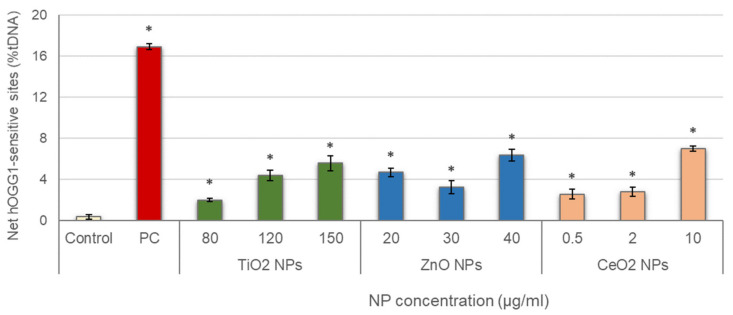
Results of oxidative DNA damage induced in salivary leucocytes exposed to TiO_2_, ZnO and CeO_2_ NPs. PC: positive control (335 µg/mL KBrO_3_ for 1 h). Columns represent mean values and error bars indicate the standard errors. * *p* < 0.01, significant difference with regard to the control.

Results obtained in the evaluation of oxidative DNA damage induced by the three different NPs are represented in Figure 4. All treatments showed values of net hOGG1-sensitive sites significantly increased with regard to the control, and significant concentration–response relationships were also observed (TiO_2_ NPs: r = 0.921; *p* < 0.01; ZnO NPs: r = 0.738; *p* < 0.01; CeO_2_ NPs: r = 0.883; *p* < 0.01).

## 4. Discussion

As with all emerging technologies, the benefits of nanotechnology must be weighed against potential health and environmental hazards associated with their development, use and disposal. Engineered nanomaterials are used in a constantly increasing number of consumer products [22], where they may enhance strength, reactivity and durability, or provide entirely new functions. However, the development of methods to evaluate the safe use of nanomaterials and assess potential associated risks has not kept pace with their rapid commercialization [23]. Due to that, these emerging anthropogenic pollutants have increasingly become a matter of public concern on the possible impact they might have on both human health and environment. 

Since the available data on nanomaterials environmental and human exposure and associated toxicity are unfortunately limited, they do not generally allow for significant quantitative risk assessment of the newly synthesized nanomaterials. Moreover, the problem of the lack of data becomes even more complicated by the questionable suitability of the toxicity tests [11]. On this regard, in recent years, some work has focused on the use of cause-and-effect analysis to systematically describe key sources of variability for in vitro nanobioassays, including the comet assay, to support method standardization [24]. Thus, a range of potential biases or artifacts were identified in the comet assay when used for testing nanomaterials, and the use of positive reference standards, and conducting control measurements to evaluate the extent of nanomaterial interference with the assay protocol (including those performed in the current study) were encouraged.

The three metal oxide NPs that were evaluated in this study are relevant nanomaterial types produced in high tonnage and of widespread use in a number of consumer products [25]. TiO_2_ NPs are one of the most widely extended engineered nanomaterials. They are largely used in a variety of industrial and medical applications including cosmetics, sunscreens, paper products, plastics, paints, drugs, and medical orthopaedic implants. They are also one of the most profusely studied nanomaterials in terms of toxicity. Due to the strong evidence collected on their harmful potential, they were classified a decade ago as human carcinogen (Category 2B) by the International Agency for Research on Cancer [26], and a very recent European Food Safety Authority report considered TiO_2_ NPs no longer safe when used as a food additive [27]. 

ZnO NPs are broadly used in various applications including cosmetics, paints, textiles, as drug carriers and fillings in medical materials [28]. Due to their good absorptive and photocatalytic properties, these metal oxide NPs are also a good option to be employed in environmental remediation for elimination or degradation of pollutants in air or water [29]. Consequently, ZnO NPs are among the most used nanomaterials. It is well-accepted that ZnO NPs are toxic to mammalian cells in vitro and to the human lung in vivo; still, their mechanism of toxicity is improperly understood [25].

CeO_2_ NPs have also a number of applications. They are widely employed in catalysts, fuel additives and polishing agents, and as UV inhibitors on outdoor surfaces [30]. Despite the high commercial interest of these NPs, their effects on human health and the environment are not completely known and studies conducted on this topic often report conflicting results [31]. Indeed, their potential toxicity is the less addressed out of the three NPs of the present study. Due to this lack of information on their potential harmful effects, the European Agency for Safety and Health at Work has recently listed CeO_2_ NPs in the top five nanomaterials worthy of investigation as a priority [32].

Large amounts of these three NPs are released to the environment as a consequence of their widespread uses; however, their health effects are not completely identified and the potential risks associated with their exposure cannot be ruled out. Since the inhalation pathway represents the main route involved in xenobiotic systemic uptake when any type of NPs are dispersed in air, salivary leucocytes seem to be a suited biomatrix to in vitro simulate and evaluate genotoxic effects of environmental or occupational exposure to NPs. They are also easy to obtain, cost-effective, and can be collected non-invasively without requiring highly trained personnel [14]. Still, to date, they have not been used before in assessing in vitro nanotoxicity.

Physical–chemical characterization of the NPs showed that nanosized particles were successfully dispersed in leucocyte culture medium. In addition, TiO_2_ and CeO_2_ NPs size slightly increased when the NPs were dispersed in medium in comparison with water, probably due to the formation of a protein corona. On the contrary, ZnO NPs hydrodynamic size resulted considerably higher in water, suggesting a higher tendency to agglomeration in this fluid rather than in cell culture medium. Zeta potential analyses by LDV showed more negative values for the three NPs dispersed in medium than in water, agreeing with previous results in CeO_2_ [33,34], ZnO [16] and TiO_2_ [35] NPs, and suggesting that upon dispersion in the medium, serum proteins are partially adsorbed to the surface of the particles.

It has been recently demonstrated that the most widespread toxicological tests commonly used to assess the possible harmfulness of different chemical agents are not fully adequate to be applied to nanomaterials [36,37]. This is mainly due to the fact that their unique physicochemical characteristics may be also responsible for unexpected interactions with test components or detection systems of standard toxicity tests. In the particular case of the comet assay, it was previously reported that a mechanism for nanomaterials interference with the alkaline protocol, involving association to nucleoid DNA, may induce breakings in the naked DNA or affect its behavior during electrophoresis [21,38]. Consequently, during the lysis step of the comet assay, nanomaterials may interact with the unprotected DNA and cause additional damage in this macromolecule, interfering with the sensitivity of the assay and thus misleading the results. However, there is still no validated protocol for the comet assay to be applied in in vitro nanogenotoxicity evaluation. Similarly, measurement of oxidative DNA damage in cells exposed to NPs could be altered due to interactions with the DNA repair enzymes employed for specifically inducing breaks in oxidized DNA bases [39]. For these reasons, possible interferences of the tested NPs with the comet assay procedures, both standard and hOGG1-modified, were checked prior evaluating potential nanogenotoxicity of these NPs. Results obtained from these analyses showed no interference of the TiO_2_, ZnO and CeO_2_ NPs with the standard alkaline comet assay in any case, discarding any alterations of DNA migration during electrophoresis, either by inducing additional breaks into the naked DNA or inhibiting DNA migration, due to the presence of these NPs. Similarly, no interference with the ability of the hOGG1 enzyme to efficiently detect oxidative DNA damage was observed for any tested NPs. Data on possible NPs interactions with the comet assay procedure are very limited, and just a previous study from our group addressed the possible interference between NPs and hOGG1 enzyme, finding interference at the highest concentration of oleic acid-coated iron oxide (Fe_3_O_4_) NPs tested (200 µg/mL) in SH-SY5Y serum-free culture medium [40]. Magdolenova et al. [21] tested the possible interference of five different NPs, namely Fe_3_O_4_ (both oleic acid-coated and uncoated), polylactic-co-glycolic acid, TiO_2_ and SiO_2_ NPs, on standard comet assay results, and found that only oleic acid-coated Fe_3_O_4_ induced a marked level of DNA damage, demonstrating the presence of interference, agreeing with our findings that TiO_2_ NPs do not interfere with the assay. Regarding the possible influence of metal oxide NPs on the activity of DNA glycosylases, Magdolenova et al. [21] found no interference of silica (SiO_2_) NPs on formamidopyrimidine DNA glycosylase (FPG) activity. However, Kain et al. [39] reported that the presence of CeO_2_ and Co_3_O_4_ NPs may influence the activity of FPG, underestimating the measurement of oxidatively damaged DNA in A549 and BEAS-2B cells exposed to these NPs. 

Once interference was shown not to occur, the standard comet assay protocol was carried out to evaluate the suitability of salivary leucocytes to detect the potential primary DNA damage induced by the study NPs. Significant dose–response increases in genetic damage were found in the three cases. To the best of our knowledge, this is the first study in which nanogenotoxicity is addressed using leucocytes collected from salivary samples as in vitro cell model. Nevertheless, there is a number of previous studies in the literature describing similar genotoxic effects to the ones described here, in different cell types, by employing the standard alkaline comet assay, corroborating the appropriateness of salivary leucocytes for nanogenotoxicity assessment. Some examples include BEAS-2B cells [41], A549 cells [42], SH-SY5Y cells [17] or human lymphocytes [43] treated with TiO_2_ NPs, and HEK293 [44], NIH/3T3 [44], A375 [45] and SH-SY5Y [16] cells exposed to ZnO NPs. In the case of CeO_2_ NPs, the literature shows certain controversy on their potential genotoxicity. Previous studies generally demonstrated that doses higher than those employed in the present study, i.e., 40, 80 or even 200 µg/mL depending on the cell type, are required to observe genotoxic effects evaluated by the comet assay on cell lines exposed to these NPs [33,39,46]. However, similarly to our findings, Auffan et al. [47] and Könen-Adıgüzel and Ergene [48] obtained dose-dependent increases of DNA damage at very low doses of CeO_2_ NPs employing human dermal fibroblasts and peripheral blood leucocytes, respectively, treated in vitro. With regard to the oxidative DNA damage, the positive responses observed in the current experiments for the three tested NPs are in agreement with previous studies employing FPG- or hOGG1-modified comet assays for evaluating TiO_2_ NPs [17,49,50], ZnO NPs [16,19], and CeO_2_ NPs [49]. On the contrary, other studies reported no primary DNA damage [51,52] or oxidative DNA damage [19,53] after exposure to these specific NPs. This could be due to the different exposure conditions or sensitivity of the cell type tested, since these parameters are well-known to be highly relevant in the genotoxicity profile of nanomaterials [54,55]. However, as previously indicated, most of these studies did not discard possible interference of NPs with the comet assay procedures, which should also be considered in the interpretation of the results obtained. According to our data, salivary leucocytes respond to even low CeO_2_ NPS concentrations, making them a valuable tool in low exposure assessments, probably consistent with real human exposure scenarios.

Future work on this topic should include quantifying NP dissolution in the cell culture medium, to determine the possible role of the released ions on the observed genotoxicity, and comparing among the different types of leucocytes, to evaluate potential differences in their sensitivity to NP induced effects.

## 5. Conclusions

Agreeing with other previous studies employing a number of different cell types, results obtained in this work showed an increase of both primary and oxidative damage induced by TiO_2_, ZnO and CeO_2_ NPs. These data support the use of salivary leucocytes as a proper and sensitive biological sample for in vitro nanogenotoxicity studies, as well as reinforce the importance of testing the suitability of standard toxicology protocols for risk assessment of nanomaterials exposure. Moreover, our data contribute to increase the knowledge on the impact of metal oxide NPs on human health, as well as support the need of a proper regulation of the use of nanomaterials in consumer products and biomedical applications.

## Figures and Tables

**Table 1:** Characterization of the NPs used in this study.

NPs	Particle Size ^a^ (nm)	Hydrodynamic Diameter (nm) ^b^ (DLS)		Zeta Potential (mV) ^b^ (LDV)	
		Water	Medium	Water	Medium
TiO_2_	25 (TEM)	199.1 ± 2.6	264.7 ± 28.2	22.8 ± 3.5	−10.6 ± 0.4
ZnO	100 (BET)	485.6 ± 26.3	199.1 ± 37.8	14.4 ± 1.7	−10.7 ± 0.7
CeO_2_	18 (BET)	175.3 ± 10.2	197.9 ± 69.4	9.9 ± 2.5	−10.4 ± 0.9

BET: Brunauer–Emmett–Teller; DLS: dynamic light scattering; LDV: laser Doppler velocimetry; TEM: transmission electronic microscopy. ^a^ Provided by the commercial supplier, ^b^ mean ± standard deviation.

## Data Availability

The data presented in this study are available on request from the corresponding author.
